# Exosomes derived from circRNA Rtn4-modified BMSCs attenuate TNF-α-induced cytotoxicity and apoptosis in murine MC3T3-E1 cells by sponging miR-146a

**DOI:** 10.1042/BSR20193436

**Published:** 2020-05-26

**Authors:** Guijun Cao, Xianqing Meng, Xiaodong Han, Jinhua Li

**Affiliations:** 1 Spinal Surgery, Affiliated Hospital of Jining Medical University, Jining, China; 2 Spine and Joint Surgery, Zoucheng People’s Hospital, Shandong Province, Jining, China; 3 Department of Orthopaedics, Sishui People’s Hospital, Shandong Province, Jining, China

**Keywords:** bone marrow-derived mesenchymal stromal cell, circular RNA Rtn4, exosomes, miR-146a, tumor necrosis factor-alpha

## Abstract

Osteoporosis is the most common and complex skeletal disorder worldwide. Exosomes secreted by bone marrow-derived mesenchymal stromal cells (BMSCs) are considered as an ideal seed source for bone tissue regeneration. However, the role of exosomes secreted by BMSCs (BMSCs-Exos) in osteoporosis and its underlying mechanisms remain unclear. In the present study, the expression of microRNA (miRNA)-146a and circular RNA (circRNA) Rtn4 (circ-Rtn4) was evaluated by quantitative real-time polymerase chain reaction (qRT-PCR), and their protein expression was determined by Western blotting. Enzyme-linked immunosorbent assay was performed to detect caspase-3 activity. Cell viability and apoptosis were assessed using 3-(4,5-Dimethylthiazol-2yl-)-2,5-diphenyl tetrazolium bromide (MTT) assay and flow cytometry analysis, respectively. Luciferase reporter assay was exploited for target validation. Results showed that tumor necrosis factor-α (TNF-α) dose-dependently increased miR-146a expression, inhibited cell viability, and promoted cell apoptosis, as indicated by increased caspase-3, cleaved caspase-3, and Bcl-2-associated X protein (Bax) expression as well as caspase-3 activity. However, miR-146a silencing or co-culture with BMSCs-Exos blocked these effects. Moreover, co-culture with exosomes-derived from circ-Rtn4-modified BMSCs (Rtn4-Exos) attenuated TNF-α-induced cytotoxicity and apoptosis in MC3T3-E1 cells, as evidenced by the decrease in caspase-3, cleaved caspase-3, and Bax protein expression and caspase-3 activity. In addition, miR-146a was identified as a target of circ-Rtn4, and Rtn4-Exos exerted their function in TNF-α-treated MC3T3-E1 cells by sponging miR-146a. Hence, our findings suggested that Rtn4-Exos attenuated TNF-α-induced cytotoxicity and apoptosis in murine MC3T3-E1 cells by sponging miR-146a, suggesting that Rtn4-Exos may serve as novel candidates for treating osteoporosis.

## Introduction

Osteoporosis is the most common and complex skeletal disorder worldwide [1]. Mechanistically, osteoporosis results from altered normal bone remodeling, including increased osteoclast activity and reduced osteoblast generation [2]. Therefore, promotion of osteoblast proliferation and inhibition of osteoblast apoptosis appear to be promising strategies for the prevention and clinical treatment of osteoporosis. Research on bone marrow-derived mesenchymal stromal cell (BMSC)-based therapies for bone disorders, including osteoporosis, has been promising [3]. Exosome, a membrane vesicle (40–150 nm in diameter) secreted by various cell types, acts as an intercellular messenger to regulate cellular function by enabling cell-to-cell transfer of biologically active molecules, such as circular RNAs (circRNAs), microRNAs (miRNAs), or messenger RNAs (mRNAs) [4,5]. Meanwhile, exosomes derived from BMSCs (BMSCs-Exos) have been reported to regulate the proliferation and apoptosis of osteoblasts, thereby indirectly promoting bone repair and improving osteoporosis [6]. However, the effects of BMSCs-Exos on tumor necrosis factor-α (TNF-α)-induced cytotoxicity and apoptosis, and the underlying mechanisms remain unknown.

circRNA, a kind of non-coding RNA generated by backsplicing, is characterized by covalently closed loop structures with neither 5′–3′ ends nor poly (A) tails [7]. Increasing research suggests that circRNAs are now considered as key regulators of cellular function instead of ‘functionless byproducts of aberrant RNA splicing’ [8]. Recently, several studies have reported that circRNA dysfunction is linked with the development of osteoporosis [9]. circRNA Rtn4 (circ-Rtn4), comprising exons 2 and 3 of the *RTN4* gene, has been shown to play a key regulatory role in the transformation of human bronchial epithelial cells induced by benzo(a)pyrene [10,11]. However, little is known about the role of circ-Rtn4 in osteoporosis.

In the present study, we aimed to determine the effects of BMSCs-Exos on TNF-α-induced cytotoxicity and apoptosis in murine MC3T3-E1 cells, and explore the underlying mechanisms. Our results showed that exosomes derived from circ-Rtn4-modified BMSCs (Rtn4-Exos) inhibited TNF-α-induced cytotoxicity and apoptosis in murine MC3T3-E1 cells via regulating miR-146a expression, thus indicating that Rtn4-Exos may serve as novel agents for the treatment of osteoporosis.

## Materials and methods

### BMSC culture

The present study was conducted at the Affiliated Hospital of Jining Medical University. All experimental procedures were performed in strict accordance with the guidelines of the Institutional Animal Ethics Committee of the Affiliated Hospital of Jining Medical University. Ethical approval was obtained from the Animal Ethics Committee before the study. BMSCs were isolated from bone marrow aspirates from 2–3-week-old C57BL/6 mice (SLAC, Shanghai, China) as previously described [12]. Mice were immediately killed by cervical dislocation. BMSCs were obtained from the femurs of C57BL/6 mice by flushing out the bone marrow with low-glucose Dulbecco’s modified Eagle’s medium (L-DMEM). Next, the cells were filtered through a 70-mm filter, resuspended in Hank’s Balanced Salt Solution, and cultured in L-DMEM containing 10% fetal bovine serum (FBS; Solarbio, Beijing, China) and 1% penicillin/streptomycin (Solarbio) at 37°C and 5% (v/v) CO_2_. The medium was changed every 2 days. At approximately 80–90% confluency, the cells were digested in 0.25% trypsin with 1 mM EDTA and the medium was replaced. After changing the medium twice, BMSCs were collected for following experiments.

To overexpress circ-Rtn4, the full sequence of circ-Rtn4 was subcloned into pcDNA-3.1 vector (Invitrogen, Carlsbad, CA, U.S.A.) to generate pcDNA-circ-Rtn4 constructs. For stable transduction of pcDNA-circ-Rtn4 or pcDNA-3.1 (negative control, NC), pcDNA-circ-Rtn4 or NC was transfected into BMSCs using Lipofectamine 2000 (Invitrogen) as per the manufacturer’s instructions.

### Isolation of exosomes

At 48 h post transfection, exosomes were isolated from the supernatant of BMSC culture transfected with pcDNA-circ-Rtn4 or NC (Rtn4-Exos or NC-Exos, respectively) using a Total Exosome Isolation kit (Invitrogen), according to the manufacturer’s recommendations. All experimental procedures were performed as described previously [13].

### Exosome uptake assay

BMSCs-Exos were labeled with the red fluorescent dye PKH26 (Sigma–Aldrich, St. Louis, MO, U.S.A.) according to the manufacturer’s instructions. Briefly, isolated exosomal pellets were resuspended in 1 ml of diluent C to prepare the BMSCs-Exos solution. Next, 6 µl of PKH26 was added into 1 ml diluent C to prepare the PKH26 solution. The BMSCs-Exos and PKH26 solutions were gently mixed for 30 s by pipetting, and then 5 ml of 1% bovine serum albumin was added to bind the excess dye. The PKH26-labeled BMSCs-Exos were centrifuged at 120000×***g*** for 2 h at 4°C, rinsed with phosphate buffered saline (PBS), and resuspended in complete culture medium. The PKH26-labeled BMSCs-Exos solution was then added into MC3T3-E1 cells and incubated for 24 h at 37°C in a humidified incubator containing 5% CO_2_. MC3T3-E1 cells were rinsed with PBS and fixed with 4% formaldehyde at 25°C for 10 min. The nuclei were stained with 1.5 µg/ml of 4′,6-diamidino-2-phenylindole (DAPI; dissolved in PBS; Sigma–Aldrich) for 3 min at room temperature. Cells were visualized by fluorescence under a confocal microscope (Olympus, Tokyo, Japan).

### MC3T3-E1 cell culture and treatment

Murine MC3T3-E1 cells were obtained from American Type Culture Collection (ATCC, Rockville, MD, U.S.A.) and grown in α-modified essential medium (Gibco, Carlsbad, CA, U.S.A.) containing 10% FBS at 37°C and 5% (v/v) CO_2_.

Anti-miR-146a, anti-miR-NC, miR-146a, and miR-NC were purchased from GenePharma (Shanghai, China). MC3T3-E1 cells were transfected with corresponding plasmids, followed by treatment with indicated doses of TNF-α (0, 2.5, 5, and 10 nM). After 48 h, MC3T3-E1 cells were co-cultured with BMSCs-Exos for 48 h.

### Cell viability assay

The 3-(4,5-Dimethylthiazol-2yl-)-2,5-diphenyl tetrazolium bromide (MTT; Solarbio) assay was performed to detect cell viability. Briefly, MC3T3-E1 cells were trypsinized, suspended in culture medium, seeded in 96-well plates, and cultured for 24 h at 37°C in 5% (v/v) CO_2_. After rinsing with PBS, MC3T3-E1 cells were treated with the indicated doses of TNF-α, with or without co-culture with exosomes derived from different BMSCs. At 48 h after treatment, MTT solution was added into each well and incubated for 4 h. Following this, cells were incubated with a formazan-dissolving solution on a shaker for 10 min in the dark. Cell viability was detected by measuring the absorbance of each well at 490 nm.

### Flow cytometry

The apoptosis of MC3T3-E1 cells was evaluated by flow cytometry using Annexin V-FITC/propidium iodide (PI) Apoptosis Detection Kit (CWBio, Beijing, China). After treatment, MC3T3-E1 cells were collected, rinsed with PBS, and suspended in Annexin-binding buffer. Thereafter, cells were gently mixed with Annexin V/FITC and PI solution in the dark for 15 min. PBS was then added into each well, and the apoptosis of MC3T3-E1 cells was detected by flow cytometry.

### Detection of caspase-3 activity

Detection of caspase-3 activity was carried out using a Caspase-3 kit (Cell Signaling Technology, Beverly, MA, U.S.A.) to assess the induction of apoptosis post treatment. In brief, MC3T3-E1 cells were collected and lysed using RIPA buffer. Next, lysed proteins were quantified using the BCA protein assay kit (Pierce, Rockford, IL, U.S.A.). Reagents were added as per the manufacturer’s instructions, and the activity of caspase-3 was determined by measuring the absorbance at 450 nm.

### Luciferase reporter assay

The wild-type (WT) sequence of circ-Rtn4 containing the miR-146a binding sites and the mutant (MUT) sequence were subcloned into pmirGLO vector (Promega, Madison, WI, U.S.A.), generating WT-circ-Rtn4 or MUT-circ-Rtn4 constructs, respectively. HEK293 and MC3T3-E1 cells were co-transfected with miR-146a or miR-NC and circ-Rtn4-WT or circ-Rtn4-MUT. Following this, cells were cultured for 24 h, and then tested for luciferase activity using a Dual-Luciferase Reporter Assay kit (Promega).

### Western blot analysis

Whole-cell lysates were prepared from MC3T3-E1 cells post treatment, and then subjected to Western blotting. Proteins were separated on 14% sodium dodecyl sulfate/polyacrylamide gels, followed by transfer on to polyvinylidene fluoride membranes. After blocking with 5% skim milk, the membranes were probed with primary antibodies against caspase-3 (Cell Signaling Technology), cleaved caspase-3 (Cell Signaling Technology), Bcl-2-associated X protein (Bax; Cell Signaling Technology), CD9 (Boster, Wuhan, China), CD81 (Boster), CD9 (Boster), Alix (Boster), and β-actin (Cell Signaling Technology), followed by incubation with horseradish peroxidase–conjugated secondary antibody (Boster). The bands were detected by chemiluminescence (Boster) and analyzed using ImageJ software (NIH, Rockville, MD, U.S.A.).

### Quantitative real-time polymerase chain reaction

RNA isolation was performed using the RNeasy Mini Kit (Qiagen, Valencia, CA, U.S.A.) according to the manufacturer’s protocol. For detection of miR-146a expression, cDNA was prepared using the TaqMan® MicroRNA Reverse Transcription Kit (Applied Biosystems, Foster City, CA, U.S.A.), followed by quantitative real-time polymerase chain reaction (qRT-PCR) analysis in conjunction with TaqMan® MicroRNA Assay (Applied Biosystems). *U6* was employed as the housekeeping gene. Meanwhile, One Step TB Green™ PrimeScript™ RT-PCR Kit (Takara, Dalian, China) was used to determine the expression of circ-Rtn4, using glyceraldehyde 3-phosphate dehydrogenase (GAPDH) as a loading control. Data were analyzed using the 2^−ΔΔ*C*_t_^ method. The gene-specific primers used were as follows: circ-Rtn4, forward: 5′-AGT ACT TAC GAA AGA AGC AGA GG-3′ and reverse: 5′-GTA TCA CAG GCT CAG ATG CAG-3′; GAPDH, forward: 5′-CGT GTT CCT ACC CCC AAT GT-3′ and reverse: 5′-TGT CAT CAT ACT TGG CAG GTT TCT-3′.

### Statistical analysis

Statistical analysis was performed with SPSS 20.0 software (IBM, Armonk, NY, U.S.A.). Normal distribution of data was assessed by Shapiro–Wilk test. Normally distributed data were expressed as the mean ± standard error of the mean. Comparison between two groups was carried out using Student’s *t* test, while comparisons between multiple groups were performed using one-way analysis of variance (ANOVA) with Tukey’s post-hoc test. *P*<0.05 was considered to be statistically significant.

## Results

### TNF-α dose-dependently increased the expression of miR-146a, decreased the viability an,d promoted the apoptosis of MC3T3-E1 cells

**Figure 1:**
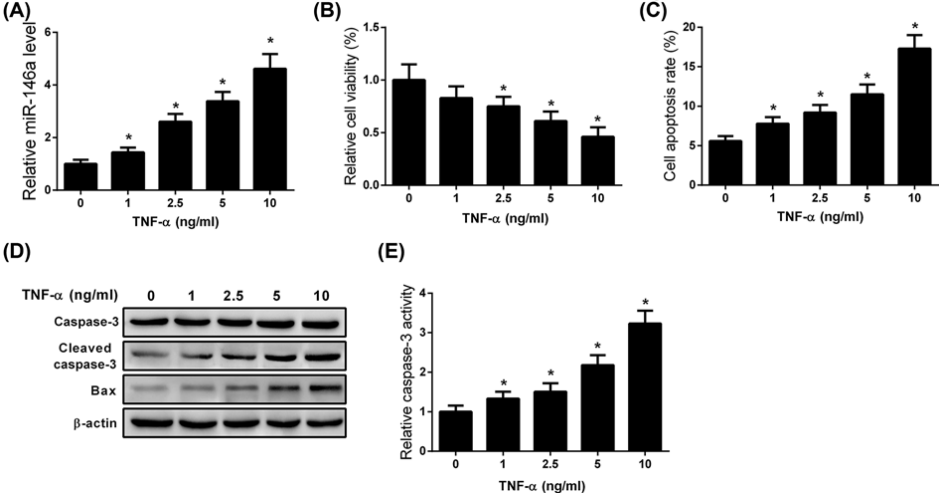
TNF-α dose-dependently increases miR-146a expression, decreases the viability, and promotes the apoptosis of MC3T3-E1 cells MC3T3-E1 cells were treated with different doses of TNF-α for 48 h. (**A**) Determination of miR-146a expression in TNF-α-treated MC3T3-E1 cells. (**B**) Cell viability was determined using the MTT assay. (**C**) Flow cytometry analysis of apoptosis in TNF-α-treated MC3T3-E1 cells. (**D**) Western blot analysis of the apoptotic proteins, caspase-3, cleaved caspase-3, and Bax in MC3T3-E1 cells treated with TNF-α. (**E**) The activity of caspase-3 was evaluated. All experiments were independently repeated three times. The caspase-3 activity and MTT assays were performed in triplicate. The differences among multiple groups were determined using one-way ANOVA test. *n*=3. *P<0.05.

We first analyzed the effect of TNF-α on MC3T3-E1 cells. As shown in Figure 1A, miR-146a expression was dose-dependently increased following TNF-α treatment. Further, treatment of MC3T3-E1 cells with TNF-α reduced cell viability and promoted apoptosis in a dose-dependent manner (Figure 1B,C). In addition, we found that TNF-α treatment dose-dependently increased cleaved caspase-3 and Bax expression as well as caspase-3 activity (Figure 1D,E).

### Down-regulation of miR-146a attenuated the cytotoxic effect of TNF-α on MC3T3-E1 cells

**Figure 2:**
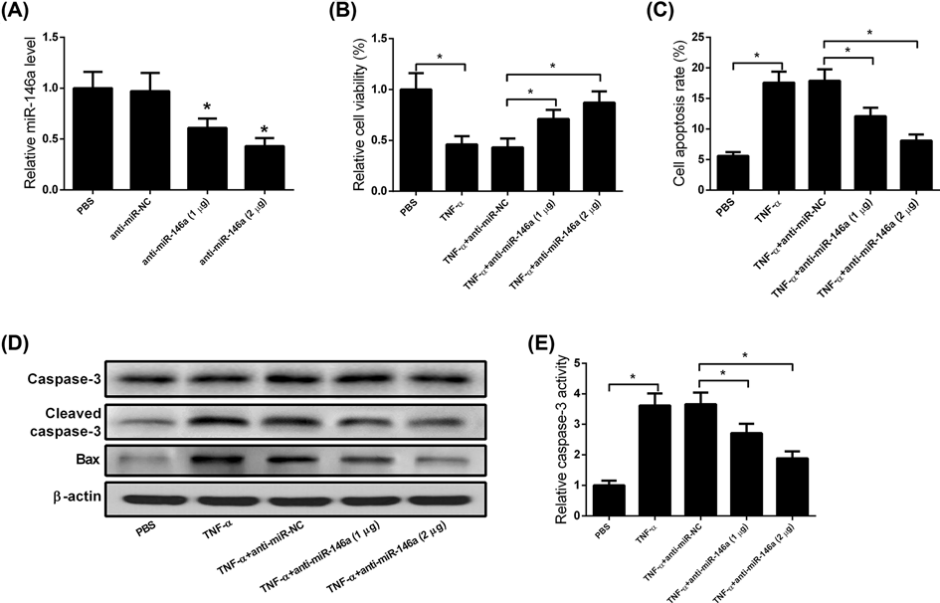
Down-regulation of miR-146a attenuates the cytotoxic effect of TNF-α on MC3T3-E1 cells (**A**) Evaluation of miR-146a expression in anti-miR-NC-, anti-miR-146a (1 μg)-, or anti-miR-146a (2 μg)-transfected MC3T3-E1 cells. (**B**) MC3T3-E1 cells were transfected with anti-miR-NC, anti-miR-146a (1 μg), or anti-miR-146a (2 μg), and then treated with TNF-α (5 ng/ml). At 48 h post-treatment, cells were subjected to MTT assay. Results revealed that down-regulation of miR-146a attenuated the cytotoxic effect of TNF-α on MC3T3-E1 cell viability. (**C**) Flow cytometry analysis showed that TNF-α-induced apoptosis was blocked by miR-146a silencing. (**D**) Western blot analysis showed that down-regulation of miR-146a blocked TNF-α-induced cleaved caspase-3 and Bax expression. (**E**) ELISA data showed that induction of caspase-3 activity by TNF-α was blocked by miR-146a knockdown. All experiments were independently repeated three times. The caspase-3 activity and MTT assays were performed in triplicate. The differences among multiple groups were determined using one-way ANOVA test. *n*=3. *P<0.05.

Since miR-146a was up-regulated in TNF-α-treated MC3T3-E1 cells, we next knocked down miR-146a expression using anti-miR-146a to explore its role in TNF-α-induced cytotoxicity. As expected, miR-146a expression was obviously reduced in anti-miR-146a-transfected MC3T3-E1 cells compared with that in anti-miR-NC-transfected cells (Figure 2A). Results of MTT assay showed that transfection of MC3T3-E1 cells with anti-miR-146a attenuated TNF-α-induced inhibition of cell viability and induction of apoptosis (Figure 2B,C). In line with this result, TNF-α-induced increase in cleaved caspase-3 and Bax expression was inhibited by silencing of miR-146a (Figure 2D). Additionally, ELISA results showed that TNF-α-induced caspase-3 activity was suppressed following miR-146a knockdown (Figure 2E).

### BMSCs-Exos mitigated TNF-α-induced cytotoxicity and apoptosis in MC3T3-E1 cells

**Figure 3:**
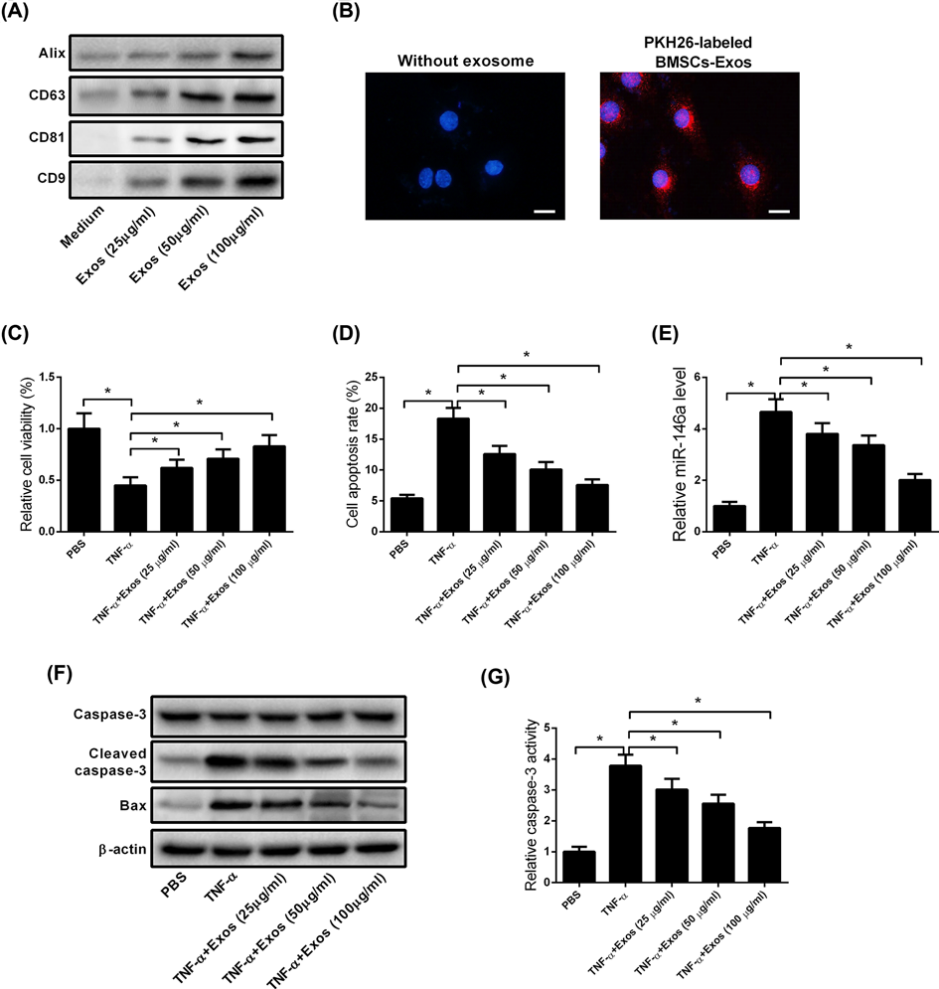
BMSCs-Exos mitigate TNF-α-induced cytotoxicity and apoptosis in MC3T3-E1 cells (**A**) Western blot analysis showed that BMSCs-Exos were positive for CD63, CD9, CD81, and Alix. (**B**) The exosome uptake assay was performed to assess the uptake of PKH26-labeled exosomes into recipient MC3T3-E1 cells. Red: PKH26-labeled BMSCs-Exos. Blue: nuclei. Scale bar = 20 μm. (**C**) MC3T3-E1 cells were treated with TNF-α (5 ng/ml) and BMSCs-Exos (0, 25, 50 and 100 μg/ml) and then subjected to cell viability testing. Results showed that BMSCs-Exos dose-dependently blocked TNF-α-induced inhibition of cell viability. (**D**) Flow cytometry analysis of MC3T3-E1 cells treated with TNF-α and BMSCs-Exos. The results showed that BMSCs-Exos dose-dependently mitigated TNF-α-induced increase in cell apoptosis. (**E**) qRT-PCR analysis showed that TNF-α-induced increase in miR-146a expression was blocked when MC3T3-E1 cells were co-cultured with BMSCs-Exos. (**F**) Western blot analysis showed that BMSCs-Exos dose-dependently blocked TNF-α-induced cleaved caspase-3 and Bax expression. (**G**) ELISA data showed that BMSCs-Exos inhibited TNF-α-induced caspase-3 activity. All experiments were independently repeated three times. The caspase-3 activity and MTT assays were performed in triplicate. The differences among multiple groups were determined using one-way ANOVA test. *n*=3. *P<0.05.

To examine the regulatory role of BMSCs-Exos, exosomes were isolated from the supernatants of BMSC cultures and confirmed by Western blotting. Results showed that CD63, CD9, CD81, and Alix were highly expressed in BMSCs-Exos, indicating that the isolated exosomes were in agreement with the current standard and could be utilized for following experiments (Figure 3A). At 24 h after co-culture with PKH26-labeled BMSCs-Exos, a red fluorescence was observed in MC3T3-E1 cells, indicating the uptake of PKH26-labeled exosomes into the recipient MC3T3-E1 cells (Figure 3B). Next, after treatment of MC3T3-E1 cells with TNF-α (5 ng/ml) and BMSCs-Exos (0, 25, 50, and 100 μg/ml), we examined cell viability and apoptosis using MTT assay and flow cytometry, respectively. The results indicated that BMSCs-Exos dose-dependently suppressed TNF-α-induced inhibition of cell viability (Figure 3C) and increase in apoptosis (Figure 3D) in MC3T3-E1 cells. Notably, we also found that up-regulation of miR-146a induced by TNF-α was inhibited when MC3T3-E1 cells were co-cultured with BMSCs-Exos (Figure 3E). Consistent with the flow cytometry data, TNF-α-induced cleaved caspase-3 and Bax expression was blocked following co-culture with BMSCs-Exos (Figure 3F). Furthermore, BMSCs-Exos inhibited TNF-α-induced caspase-3 activity in a dose-dependent manner (Figure 3G).

### Rtn4-Exos attenuated TNF-α-induced cytotoxicity and apoptosis in murine MC3T3-E1 cells

**Figure 4:**
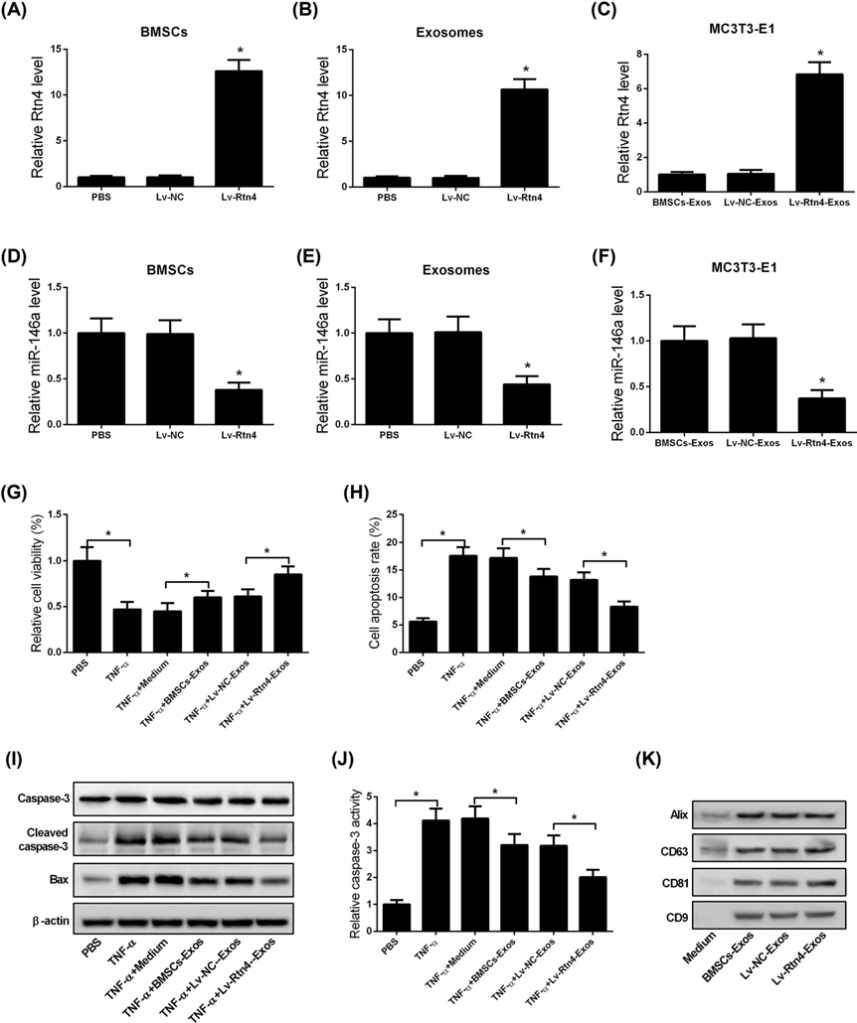
Rtn4-Exos prevent TNF-α-induced cytotoxicity and apoptosis in murine MC3T3-E1 cells BMSCs were transfected with NC or pcDNA-circ-Rtn4, and their exosomes were isolated. (**A,B**) The expression of circ-Rtn4 was measured in NC- or pcDNA-circ-Rtn4-transfected BMSCs and their exosomes using qRT-PCR. (**C**) MC3T3-E1 cells were co-cultured with Rtn4-Exos or NC-Exos, and tested for circ-Rtn4 expression using qRT-PCR. (**D,E**) Evaluation of miR-146a expression in NC- or pcDNA-circ-Rtn4-transfected BMSCs and their exosomes using qRT-PCR. (**F**) qRT-PCR analysis of miR-146a expression in MC3T3-E1 cells treated with Rtn4-Exos or NC-Exos. (**G**) MC3T3-E1 cells were treated with TNF-α, followed by co-culture with Rtn4-Exos or NC-Exos. The viability of MC3T3-E1 cells was evaluated using MTT assay. (**H**) Flow cytometry analysis to evaluate cell apoptosis in MC3T3-E1 cells treated with TNF-α and exosomes from different sources. (**I**) The protein expression levels of caspase-3, cleaved caspase-3, and Bax were determined using Western blotting. (**J**) Caspase-3 activity was measured in MC3T3-E1 cells treated with TNF-α and exosomes from different sources using ELISA. (**K**) Western blot analysis of surface markers (CD63, CD81, CD9, and Alix) in exosomes. All experiments were independently repeated three times. The caspase-3 activity and MTT assays were performed in triplicate. The differences among multiple groups were determined using one-way ANOVA test. *n*=3. *P<0.05.

circRNAs have been reported to play a vital role in osteoporosis. To determine whether BMSCs package pcDNA-circ-Rtn4 into secreted exosomes, BMSCs were transfected with pcDNA-circ-Rtn4 or NC. At 48 h post transfection, we evaluated the expression of circ-Rtn4 and isolated exosomes from the supernatants. Results showed that the expression of circ-Rtn4 was significantly increased in pcDNA-circ-Rtn4-transfected BMSCs compared with that in NC-transfected BMSCs (Figure 4A). In addition, Rtn4-Exos showed increased expression of circ-Rtn4 (Figure 4B). Next, to test whether BMSCs-Exos carrying pcDNA-circ-Rtn4 could deliver circ-Rtn4 into the cells, we exposed MC3T3-E1 cells to Rtn4-Exos or NC-Exos. After 48 h, an increase in circ-Rtn4 expression was observed in MC3T3-E1 cells co-cultured with Rtn4-Exos (Figure 4C). Moreover, we found that miR-146a expression was markedly reduced in pcDNA-circ-Rtn4-transfected BMSCs, Rtn4-Exos, and MC3T3-E1 cells co-cultured with Rtn4-Exos (Figure 4D–F). To further determine whether Rtn4-Exos affected TNF-α-induced cytotoxicity and apoptosis in murine MC3T3-E1 cells, cells were treated with TNF-α and then co-cultured with Rtn4-Exos or NC-Exos. The results of MTT assay showed that co-culture of MC3T3-E1 cells with Rtn4-Exos mitigated the inhibitory effect of TNF-α on cell viability (Figure 4G). In parallel, flow cytometry analysis revealed that TNF-α-induced cell apoptosis was blocked when MC3T3-E1 cells were co-cultured with Rtn4-Exos (Figure 4H). In line with this result, the expression of cleaved caspase-3 and Bax was reduced in MC3T3-E1 cells treated with TNF-α and Rtn4-Exos, as indicated by Western blotting (Figure 4I). Additionally, compared with MC3T3-E1 cells treated with TNF-α and NC-Exos, cells treated with TNF-α and Rtn4-Exos significantly reduced caspase-3 activity (Figure 4J). Moreover, BMSCs-Exos, Rtn4-Exos, and NC-Exos were found to highly express CD63, CD9, CD81, and Alix, as confirmed by Western blot analysis (Figure 4K).

### circ-Rtn4 functioned as a sink for miR-146a

**Figure 5:**
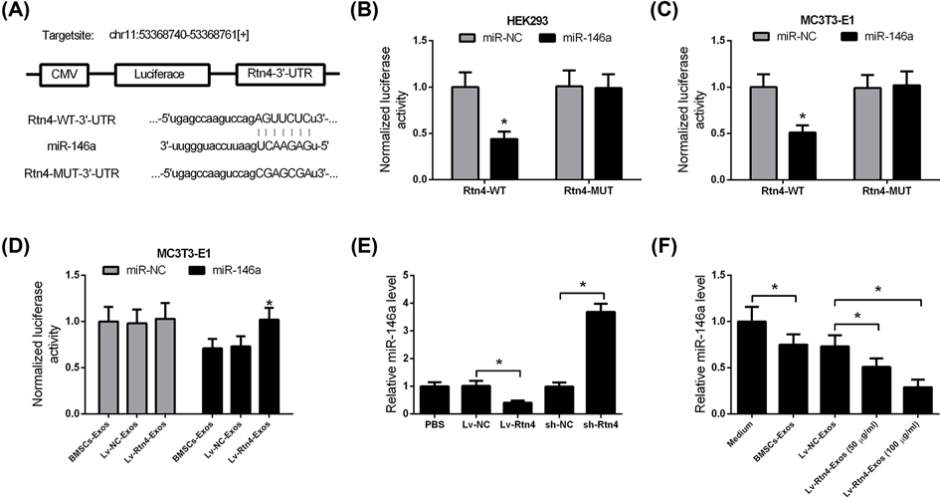
circ-Rtn4 functions as an miR-146a sponge (**A**) Schema representing the functional interaction between miR-146a and circ-Rtn4. (**B,C**) HEK293 and MC3T3-E1 cells were co-transfected with miR-146a or miR-NC and luciferase reporter containing circ-Rtn4-WT or circ-Rtn4-MUT. After 48 h, the luciferase activity was measured. (**D**) Luciferase reporters containing circ-Rtn4-WT and miR-146a or miR-NC were co-transfected into MC3T3-E1 cells in the presence of BMSCs-Exos, Lv-NC-Exos, or Lv-Rtn4-Exos. After 48 h, the luciferase activity was determined. (**E**) MC3T3-E1 cells were transfected with pcDNA-circ-Rtn4, short hairpin RNA targeting circ-Rtn4 (sh-Rtn4), or matched controls. After 48 h, the expression of miR-146a was examined using qRT-PCR. (**F**) MC3T3-E1 cells were co-cultured with BMSCs-Exos, Lv-NC-Exos, or Lv-Rtn4-Exos. After 48 h, the expression of miR-146a was examined using qRT-PCR. All experiments were independently repeated three times. The luciferase reporter gene assay was performed in triplicate. The difference between two groups was determined using Student’s *t* test. The differences among multiple groups were determined using one-way ANOVA test. *n*=3. *P<0.05.

Given that circRNAs exert their functions by sponging miRNAs, and circ-Rtn4 sequence harbors putative binding sites for miR-146a, miR-146a was predicted as a potential target of circ-Rtn4 (Figure 5A). To explore whether circ-Rtn4 functions as a sink for miR-146a, the luciferase reporter assay was carried out. In both HEK193 and MC3T3-E1 cells, overexpression of miR-146a markedly inhibited the luciferase activity of luciferase reporters containing circ-Rtn4-WT, but had no effect on the activity of reporters containing circ-Rtn4-MUT (Figure 5B,C). Compared with BMSCs-Exos and Lv-NC-Exos, Lv-Rtn4-Exos markedly abolished the inhibitory effect of miR-146a on the luciferase activity of reporters containing circ-Rtn4-WT (Figure 5D). Moreover, we found that overexpression of circ-Rtn4 decreased, while knockdown of circ-Rtn4 increased the expression of miR-146a (Figure 5E). Besides, co-culture of MC3T3-E1 cells with Rtn4-Exo decreased miR-146a expression (Figure 5F).

### Rtn4-Exos mitigated TNF-α-induced cytotoxicity and apoptosis in murine MC3T3-E1 cells by acting as an miR-146a sponge

**Figure 6:**
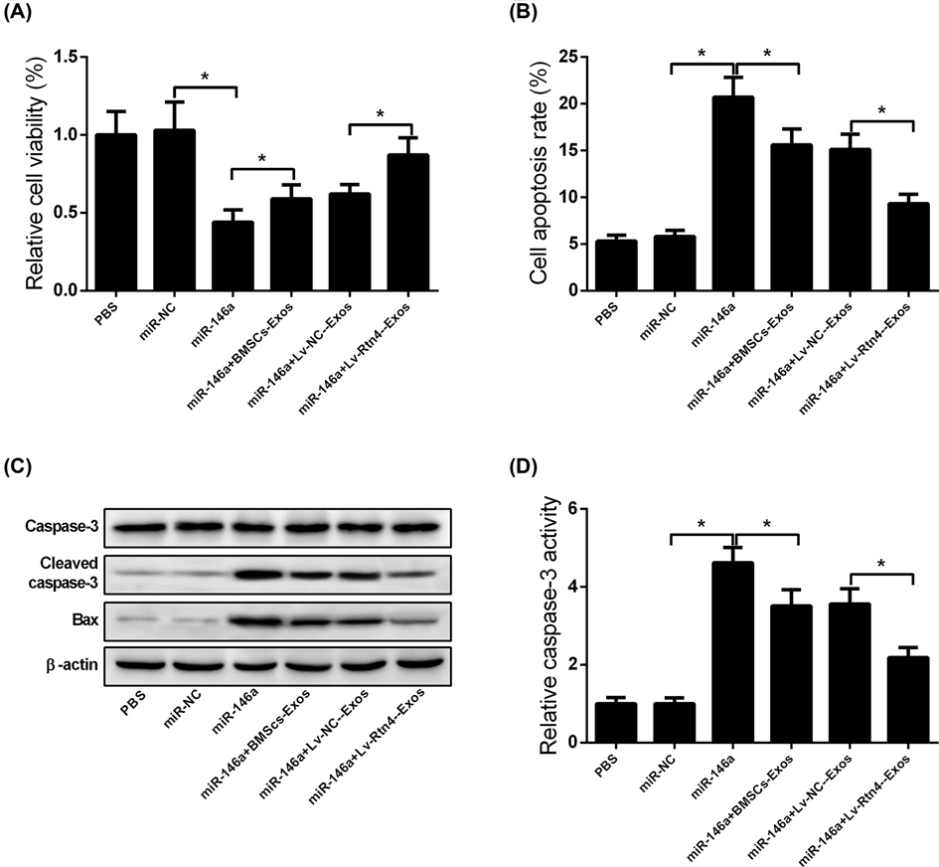
Rtn4-Exos mitigate TNF-α-induced cytotoxicity and apoptosis in murine MC3T3-E1 cells by acting as an miR-146a sponge MC3T3-E1 cells were transfected with miR-146a or miR-NC. At 48 h post transfection, MC3T3-E1 cells were treated with TNF-α and then co-cultured with BMSCs-Exos, Rtn4-Exos, or NC-Exos. (**A**) The viability of MC3T3-E1 cells was evaluated using MTT assay. (**B**) Cell apoptosis was analyzed by flow cytometry. (**C**) Comparison of caspase-3, cleaved caspase-3, and Bax expression among indicated MC3T3-E1 cells using Western blotting. (**D**) Determination of caspase-3 activity using ELISA. All experiments were independently repeated three times. The caspase-3 activity and MTT assays were performed in triplicate. The differences among multiple groups were determined using one-way ANOVA test. *n*=3. *P<0.05.

In order to confirm whether Rtn4-Exos mitigate TNF-α-induced cytotoxicity and apoptosis in murine MC3T3-E1 cells by acting as an miR-146a sponge, cells were transfected with miR-146a or miR-NC. At 48 h post transfection, MC3T3-E1 cells were treated with TNF-α and then co-cultured with BMSCs-Exos, Rtn4-Exos, or NC-Exos. As shown in Figure 6A,B, overexpression of miR-146a enhanced TNF-α-induced cytotoxicity and apoptosis in MC3T3-E1 cells, and this effect was remarkably suppressed by co-culture with BMSCs-Exos or Rtn4-Exos. Meanwhile, up-regulation of miR-146a increased cleaved caspase-3 and Bax expression as well as caspase-3 activity, as indicated by Western blotting and ELISA, respectively. However, these effects of miR-146a overexpression were mitigated following co-culture with BMSCs-Exos or Rtn4-Exos (Figure 6C,D).

## Discussion

Osteoblasts and osteoclasts are reportedly sensitive to cytokines and growth factors, which have been implicated in modulation of bone remodeling [14]. Among them, TNF-α is a multifunctional cytokine that serves as a key regulator of osteoporosis pathology [15]. TNF-α can also stimulate osteoblasts to produce other cytokines, such as interleukin (IL)-1 and IL-6, which exert direct effects on osteoclasts and promote bone resorption, resulting in bone loss [16]. Importantly, TNF-α is considered as an inducer of osteoblast apoptosis [17,18]. Further, TNF-α has been shown to inhibit the bone-forming function of osteoblasts through various mechanisms, including interaction with its receptors and death receptors [19]. Hence, it is speculated that TNF-α-induced osteoblast apoptosis might be responsible for bone loss, resulting in osteoporosis. Thus, new therapeutic strategies are urgently needed to efficiently repress TNF-α-induced cytotoxicity and apoptosis for the treatment of osteoporosis.

Exosomes have been found to be new mediators of intercellular communication. Recently, exosomes have been demonstrated to play an important role in the etiology of bone metabolic diseases, including osteoporosis [20]. In particular, exosomes are capable of influencing cell function by transferring biologically active molecules to recipient cells through membrane fusion. For example, exosomes derived from LNCaP cells were shown to increase osteoblast activity through transferring miR-375 [21]. In addition, multiple myeloma-derived epidermal growth factor receptor (EGFR) ligand amphiregulin-enriched exosomes were shown to promote EGFR pathway activation, inhibit osteoblast differentiation, and induce osteoclastogenesis [22]. BMSCs-Exos have been shown to promote the proliferation of hFOB 1.19 cells through the mitogen-activated protein kinase signaling pathway [23]. Given the role of BMSCs-Exos in bone regenerative therapy, we investigated whether BMSCs-Exos can attenuate TNF-α-induced cytotoxicity and apoptosis. Our results showed that TNF-α dose-dependently increased miR-146a expression, decreased cell viability, and promoted cell apoptosis, as indicated by increased caspase-3, cleaved caspase-3, and Bax expression as well as caspase-3 activity. However, co-culture with BMSCs-Exos blocked this effect, indicating the potential role of BMSCs-Exos in treating osteoporosis. Non-coding RNAs are selectively enriched in exosomes. In recent years, several studies showed that exosomes are involved in cellular processes through intercellular communication by carrying non-coding RNAs, such as miRNAs, lncRNAs, and circRNAs. In the present study, we focus on the effect of Rtn4-Exos on TNF-α-induced cytotoxicity and apoptosis in MC3T3-E1 cells.

To date, much attention has been focused on identifying the significance of circRNAs in human diseases. Relatively, there are few studies on the role of circRNAs in osteoporosis. In 2018, Jin et al. [24], using Illumina-based complementary DNA deep sequencing, identified that 260 circRNAs were differentially expressed (106 up-regulated and 154 down-regulated) in the peripheral blood lymphocytes of patients with postmenopausal osteoporosis relative to the healthy controls. Additional studies assessing the role of hsa_circ_0001275 in osteoporosis have reported that hsa_circ_0001275 expression is strikingly up-regulated in the peripheral blood mononuclear cells of patients with postmenopausal osteoporosis and inversely correlated with the T-score, suggesting that hsa_circ_0001275 might serve as a potential diagnostic biomarker for postmenopausal osteoporosis [25]. Nevertheless, no studies have been reported on the role of circ-Rtn4 in osteoporosis. In our study, increased expression of circ-Rtn4 was observed in pcDNA-circ-Rtn4-transfected BMSCs, Rtn4-Exos, and MC3T3-E1 cells co-cultured with Rtn4-Exos. Mechanistic investigations suggested that co-culture with Rtn4-Exos attenuated TNF-α-induced cytotoxicity and apoptosis in MC3T3-E1 cells, as evidenced by the decrease in cleaved caspase-3 and Bax protein expression and caspase-3 activity, thus suggesting that Rtn4-Exos may serve as potential candidates for the treatment of osteoporosis.

However, the mechanism by which Rtn4-Exos attenuates TNF-α-induced cytotoxicity and apoptosis in MC3T3-E1 cells has not yet been fully studied. circRNAs have been shown to function as miRNA sponges by competing with mRNAs for miRNA response elements. Through bioinformatics analysis, we found that the circ-Rtn4 sequence contained miR-145a binding sites, which have been implicated in osteoblastogenesis. Previously, a functional study assessing the role of miR-146a in osteoblasts reported that miR-146a repressed proliferation and promoted apoptosis of MC3T3-E1 cells through inhibiting B-cell lymphoma-2 [26], thus highlighting the inhibitory effect of miR-146a on osteoblasts. In osteoarthritis, miR-146a was shown to be involved in bone destruction, as miR-146a knockdown in osteoarthritic mice resulted in alleviation of articular cartilage degeneration [27]. However, the role of miR-146a in TNF-α-induced cytotoxicity and apoptosis remains elusive. Herein, our results showed that miR-146a was up-regulated in TNF-α-treated MC3T3-E1 cells and silencing of miR-146a blocked the cytotoxic effect of TNF-α on MC3T3-E1 cells. Moreover, using luciferase reporter assay, miR-146a was identified as a direct target of circ-Rtn4. More importantly, rescue experiments revealed that co-culture with Rtn4-Exos could block miR-146a overexpression-induced inhibition of cell growth in TNF-α-treated MC3T3-E1 cells, implying that Rtn4-Exos exerted their function by sponging miR-146a. Other regulatory mechanisms of BMSCs-Exos in the pathological process of osteoporosis still need further study to elucidate.

In conclusion, our findings demonstrated that TNF-α inhibited cell proliferation and induced apoptosis by up-regulating caspase-3 and Bax expression in MC3T3-E1 cells; however, these effects were abrogated by silencing of miR-146a, or co-culture with BMSCs-Exos or Rtn4-Exos. Further experiments showed that Rtn4-Exos attenuated TNF-α-induced cytotoxicity and apoptosis by sponging miR-146a, suggesting that Rtn4-Exos might serve as promising candidates for the treatment of TNF-α-induced osteoporosis. As a limitation of the present study, we did not determine whether BMSCs-Exos have an effect on differentiation and mineralization in MC3T3-E1 cells, as well as in an *in vivo* model of osteoporosis. Hence, more efforts should be made to address these limitations in the future.

## Abbreviations

Bax: Bcl-2-associated X protein
BCA: bicinchoninic acid assay
Bcl-2: B-cell lymphoma 2
BMSC: bone marrow-derived mesenchymal stromal cell
BMSCs-Exo: exosomes derived from BMSC
circRNA: circular RNA
circ-Rtn4: circRNA Rtn4
EGFR: epidermal growth factor receptor
FBS: fetal bovine serum
GAPDH: glyceraldehyde 3-phosphate dehydrogenase
IL: interleukin
L-DMEM: low-glucose Dulbecco’s modified Eagle’s medium
miRNA: microRNA
mRNA: messenger RNA
MTT: 3-(4,5-Dimethylthiazol-2yl-)-2,5-diphenyl tetrazolium bromide
NC: negative control
PBS: phosphate buffered saline
PI: propidium iodide
RIPA: radioimmunoprecipitation assay
Rtn4: Reticulon 4
Rtn4-Exo: circ-Rtn4-modified BMSC
TNF-α: tumor necrosis factor-α
WT: wild-type
