# Expression of Concern: Exosomes derived from circRNA Rtn4-modified BMSCs attenuate TNF-α-induced cytotoxicity and apoptosis in murine MC3T3-E1 cells by sponging miR-146a

**DOI:** 10.1042/BSR20193436_EOC

**Published:** 2026-06-25

**Authors:** Guijun Cao, Xianqing Meng, Xiaodong Han, Jinhua Li

**Affiliations:** 1 Spinal Surgery, Affiliated Hospital of Jining Medical University, Jining, China; 2 Spine and Joint Surgery, Zoucheng People's Hospital, Shandong Province, Jining, China; 3 Department of Orthopaedics, Sishui People's Hospital, Shandong Province, Jining, China

**Keywords:** bone marrow-derived mesenchymal stromal cell, circular RNA Rtn4, exosomes, miR-146a, tumor necrosis factor-alpha

*Bioscience Reports* has been made aware of potential issues surrounding the scientific validity of this paper and is hence issuing a permanent Expression of Concern to notify readers.

Concerns have been raised regarding similarity with the Figure panels from Ji et al. 2016 (DOI: 10.1016/j.mce.2016.06.029). Specifically, the similarities include:

- In Figure 6C of DOI 10.1042/BSR20193436, the Caspase-3 panel appears highly similar to that of the Figure 4F Akt panel of DOI 10.1016/j.mce.2016.06.029.
- In Figure 6C of DOI 10.1042/BSR20193436, the beta-actin panel appears highly similar to that of the Figure 4F beta-action panel of DOI 10.1016/j.mce.2016.06.029.

The authors have not responded to the journal's investigation or provided raw data. As such, the article will remain published but with an associated Expression of Concern.

